# Genome-wide SNP genotyping highlights the role of natural selection in *Plasmodium falciparum *population divergence

**DOI:** 10.1186/gb-2008-9-12-r171

**Published:** 2008-12-15

**Authors:** Daniel E Neafsey, Stephen F Schaffner, Sarah K Volkman, Daniel Park, Philip Montgomery, Danny A Milner, Amanda Lukens, David Rosen, Rachel Daniels, Nathan Houde, Joseph F Cortese, Erin Tyndall, Casey Gates, Nicole Stange-Thomann, Ousmane Sarr, Daouda Ndiaye, Omar Ndir, Soulyemane Mboup, Marcelo U Ferreira, Sandra do Lago Moraes, Aditya P Dash, Chetan E Chitnis, Roger C Wiegand, Daniel L Hartl, Bruce W Birren, Eric S Lander, Pardis C Sabeti, Dyann F Wirth

**Affiliations:** 1 Broad Institute of MIT and Harvard, 7 Cambridge Center, Cambridge, MA 02142, USA; 2 Department of Immunology and Infectious Diseases, Harvard School of Public Health, 677 Huntington Ave, Boston, MA 02115, USA; 3 School for Health Studies, Simmons College, 300 The Fenway, Boston, MA 02115, USA; 4 Faculty of Medicine and Pharmacy, Cheikh Anta Diop University, BP 7325 Dakar, Senegal; 5 Departamento de Parasitologia, Instituto de Ciencias Biomedicas da USP, Av. Prof. Lineu Prestes 1374, Cidade Universitaria, 05508-900 Sao Paulo, SP, Brazil; 6 Instituto de Medicina Tropical de Sao Paulo, Universidade de Sao Paulo, Av Dr. Eneas de Carvalho Aguiar 470, 05403-907 Sao Paulo, SP, Brazil; 7 National Institute of Malaria Research, 22, Sham Nath Marg, Delhi-110054, India; 8 International Centre for Genetic Engineering and Biotechnology, Aruna Asaf Ali Marg, New Delhi-110067, India; 9 Department of Organismic and Evolutionary Biology, Harvard University, 16 Divinity Avenue, Cambridge, MA 02138, USA

## Abstract

**Background:**

The malaria parasite *Plasmodium falciparum *exhibits abundant genetic diversity, and this diversity is key to its success as a pathogen. Previous efforts to study genetic diversity in *P. falciparum *have begun to elucidate the demographic history of the species, as well as patterns of population structure and patterns of linkage disequilibrium within its genome. Such studies will be greatly enhanced by new genomic tools and recent large-scale efforts to map genomic variation. To that end, we have developed a high throughput single nucleotide polymorphism (SNP) genotyping platform for *P. falciparum*.

**Results:**

Using an Affymetrix 3,000 SNP assay array, we found roughly half the assays (1,638) yielded high quality, 100% accurate genotyping calls for both major and minor SNP alleles. Genotype data from 76 global isolates confirm significant genetic differentiation among continental populations and varying levels of SNP diversity and linkage disequilibrium according to geographic location and local epidemiological factors. We further discovered that nonsynonymous and silent (synonymous or noncoding) SNPs differ with respect to within-population diversity, inter-population differentiation, and the degree to which allele frequencies are correlated between populations.

**Conclusions:**

The distinct population profile of nonsynonymous variants indicates that natural selection has a significant influence on genomic diversity in *P. falciparum*, and that many of these changes may reflect functional variants deserving of follow-up study. Our analysis demonstrates the potential for new high-throughput genotyping technologies to enhance studies of population structure, natural selection, and ultimately enable genome-wide association studies in *P. falciparum *to find genes underlying key phenotypic traits.

## Abstract 2

An array-based SNP genotyping platform for Plasmodium falciparum is reported together with an analysis of SNP diversity in global population samples.

## Background

*Plasmodium falciparum *is the most virulent species of malaria and the primary cause of malaria-related mortality across the globe. The success of *P. falciparum *as a pathogen derives in part from its high levels of genetic diversity [1-4], diversity that endows the parasite with the evolutionary agility to rapidly develop resistance to a series of drugs developed for its control [5], to thwart the development of effective vaccines [6], and to efficiently evade immune responses [7-9]. Large-scale genotyping of *P. falciparum *will improve understanding of these capabilities, and will permit wide-ranging investigation of the parasite's biology, including population structure and history, outcrossing and recombination frequency and instances of natural selection, and inform effective intervention strategies. As a target for genotyping, *P. falciparum *has the advantage that it is haploid during human stages of its life cycle, making identification of haplotypes and inference of outcrossing from patient isolates more straightforward than in primarily diploid parasites.

Prior multilocus analyses have revealed important aspects of the parasite's biology even though these studies did not encompass the entire genome. Microsatellite studies, the largest using 342 loci, have revealed geographic differences in genetic diversity and linkage disequilibrium (LD) that are correlated with the incidence of multiple infections, with diversity highest in African populations and lowest in South American populations [10], as well as evidence for multiple, recent selective sweeps [11]. A recent study of 183 single nucleotide polymorphisms (SNPs) on chromosome 3 came to similar conclusions, finding geographic variation in effective recombination rate, frequency of out-crossing and strong population structure at the continental scale [12].

Recent genome-wide surveys of genomic variation in *P. falciparum *have uncovered tens of thousands of new SNPs, indels, and structural variants [2-4,13]. This resource opens the door to comprehensive genotyping analyses, including genome-wide scans for natural selection and genome-wide association studies for genetic loci underlying phenotypes like drug resistance and virulence. Inferences from genome wide analyses of diversity are less subject to the inherent biases associated with analyses of individual antigenic loci, which may be targets of strong natural selection, and promise to more accurately reflect overall patterns of genetic variation in *P. falciparum*. The first step needed is to develop high-throughput genotyping technology that can take advantage of the extensive new diversity data.

In this manuscript we report the development of a 3,000 SNP Affymetrix genotyping array and initial biological observations resulting from its deployment with 76 globally distributed parasite isolates. We demonstrate the ability of this platform to contend with the extremely high (approximately 81%) A/T composition of the *P. falciparum *genome, with mixed parasite genotypes, and with human DNA contamination. Using SNP data collected from the array, we confirm with greater resolution differences in the level of genomic diversity between African, Asian, and American populations of *P. falciparum*, document genetic differentiation of those continental populations as well as the presence of structure within non-African populations, and explore the important roles of natural selection and recombination in sculpting genetic variation within malaria populations. We anticipate that a scaled-up genotyping array based on this now-proven technology will usher in fundamental new insights into basic malaria biology as well as novel disease intervention strategies.

## Results

### Array assessment

The genotyping array employs a standard Affymetrix 500 K array design, utilizing a total of 56 probes arrayed in 14 quartets to interrogate each SNP. Mismatch probes were utilized in order to evaluate the effectiveness of the Dynamic Modeling (DM) calling algorithm for the A/T-rich *P. falciparum *genome. We included 2,153 SNPs from chromosome 7 on the array to assess patterns of LD near a known selective sweep [11], as well as 847 SNPs selected from other genomic locations distributed across all 14 chromosomes. We hybridized a total of 108 samples to the Affymetrix array to assess its performance. These samples included the 16 *P. falciparum *strains we used for SNP ascertainment [3], in order to assess genotyping accuracy as well as genotyping call rate. We applied three SNP calling algorithms to the data: DM, BRLMM (Bayesian Robust Linear Modeling using Mahalanobis Distance), and BRLMM-P. The BRLMM-P algorithm yielded the highest call rate and concordance with known genotypes. The average BRLMM-P call rate for the 16 strains used for SNP ascertainment was 91% (2,732 assays out of 3,000), similar to what was observed for early generations of Affymetrix human SNP arrays [14]. Overall concordance with known genotype for all genotype calls was also 91%, indicating that the array is able to accommodate the extremely high A/T composition of the *P. falciparum *genome. A number of assays consistently yielded only the major (most common) SNP allele, suggesting either that those assays were faulty or that the genomic positions the assays targeted were incorrectly identified as polymorphic. Removal of these uninformative assays as well as assays that gave an incorrect genotype call for one or more samples yielded a set of 1,638 assays that achieved perfect concordance with known genotypes for both the major and minor alleles in the 16 samples used for SNP ascertainment. A histogram of DM, BRLMM, and BRLMM-P call rates for this validated set of 1,638 SNPs in the ascertainment sample set is illustrated in Additional data file 1. Replicate hybridization data (not shown) for the HB3 and 3D7 samples indicate that overall genotyping consistency is 99.9%. Results of experiments to test array performance in the presence of human DNA or DNA from mixed malaria infections are included in Additional data files 2-4.

### Population samples

We hybridized a global collection of parasite isolates to the array to examine population structure, SNP diversity, and LD in the *P. falciparum *genome. This collection is summarized in Table 1, and includes 23 samples from Thailand, 20 samples from Senegal, 11 samples from Brazil, 8 samples from Malawi, 2 samples from India, as well as single isolates from a variety of other African, Asian/Pacific, and Central/South American locales. As noted in Table 1, a small number of patient isolate samples are suspected to be mixtures of genetically distinct strains rather than single lineages based on the results of PCR-based assays of high frequency SNPs (data not shown). Genotyping data from these strains were excluded from all LD analyses, but included in diversity and divergence analyses. We expect the genotyping data from mixed samples to predominantly reflect the genotype of the strain present in greatest abundance, based on experimental hybridizations with formulated strain mixtures (Additional data file 3). We also hybridized DNA from *P. reichenowi*, the species most closely related to *P. falciparum*, in order to root allelic dimorphisms. All analyses were performed using a set of 1,441 assays (out of the 1,638 validated SNPs) that achieved a call rate of at least 80% among all 76 geographic or SNP ascertainment samples. A total of 874 assays of this subset of 1,441 come from chromosome 7; 281 SNPs are intergenic, 48 are intronic, 386 are synonymous coding, and 726 are nonsynonymous. Assayed genic SNPs occur in 180 genes on chromosome 7 and 487 genes from all 13 of the remaining chromosomes. The genomic coordinates (using the 3D7 PlasmoDB 5.0 assembly as reference) of all 1,441 assays used in downstream analyses are included in Additional data file 5, along with genotype information for all 76 geographic or SNP ascertainment samples. These genotyping data have also been submitted to PlasmoDB [15].

**Table 1:** Samples used for array validation and diversity analyses

Parasite line	Origin	Source	Single infection?*	Chloroquine resistant (R)/sensitive (S)
7G8	Brazil	MRA-152	Yes	
ADA-2	Brazil	Sandra do Lago Moraes	Yes	
9_411	Brazil	Alejandro Miguel Katzin	Yes	
10_54	Brazil	Alejandro Miguel Katzin	Yes	
608_88	Brazil	Alejandro Miguel Katzin	Yes	
36_89	Brazil	Alejandro Miguel Katzin	Yes	
51	Brazil	Alejandro Miguel Katzin	Yes	
JST	Brazil	Sandra do Lago Moraes	Yes	
356_89	Brazil	Alejandro Miguel Katzin	Yes	
330_89	Brazil	Alejandro Miguel Katzin	Yes	
207_89	Brazil	Alejandro Miguel Katzin	Yes	
FCC-2/Hainan	China	MRA-733	Yes	S
Santa Lucia (El Salvador)	El Salvador	MRA-362	Yes	
RO-33	Ghana	MRA-200	Yes	S
HB3	Honduras	MRA-155	Yes	
IGHC14	India	Aditya Dash, Chetan Chitnis	Yes	
RAJ116	India	Aditya Dash, Chetan Chitnis	Yes	
Dd2	Indochina/Laos	MRA-156	Yes	R
KMWII	Kenya	MRA-821	Yes	
CF04.008 10B	Malawi	Dan Milner	Yes	
CF04.008 12G	Malawi	Dan Milner	Yes	
CF04.008 1F	Malawi	Dan Milner	Yes	
CF04.008 2G	Malawi	Dan Milner	Yes	
CF04.008 7H	Malawi	Dan Milner	Yes	
CF04.009 6D	Malawi	Dan Milner	Yes	
CF04.010 10B	Malawi	Dan Milner	Yes	
Malawi CF04.008	Malawi	Dan Milner	No	
Malayan Camp R+	Malaysia	MRA-330	Yes	S
3D7	Netherlands	MRA-151	Yes	S
D10	Papua New Guinea	MRA-201	Yes	S
Senegal V34.04	Senegal	J Daily	Yes	S
Senegal P31.01	Senegal	J Daily	Yes	S
Senegal P51.02	Senegal	M Duraisingh	Yes	R
Senegal V35.04	Senegal	J Daily	Yes	S
Senegal P18.02	Senegal	M Duraisingh	No	
Senegal P08.04	Senegal	M Duraisingh	Yes	S
Senegal P26.04	Senegal	M Duraisingh	Yes	R
Senegal P27.02	Senegal	M Duraisingh	Yes	S
Senegal P60.02	Senegal	M Duraisingh	Yes	R
Senegal Thi10.04	Senegal	M Duraisingh	No	
Senegal Thi26.04	Senegal	M Duraisingh	Yes	R
Senegal V56.04	Senegal	J Daily	No	
Senegal P05.02	Senegal	M Duraisingh	Yes	R
Senegal P06.02	Senegal	M Duraisingh	No	
Senegal P09.04	Senegal	M Duraisingh	Yes	S
Senegal P11.02	Senegal	M Duraisingh	No	
Senegal P19.04	Senegal	M Duraisingh	Yes	S
Senegal Thi15.04	Senegal	M Duraisingh	Yes	
Senegal Thi28.04	Senegal	M Duraisingh	Yes	R
Senegal V42.05	Senegal	Dan Milner	Yes	S
D6	Sierra Leone	MRA-285	Yes	S
K1	Thailand	MRA-159	Yes	R
T9-94	Thailand	MRA-153	Yes	R
TM93C1088	Thailand	MRA-207	Yes	R
TM90C6B	Thailand	MRA-205	No	
TM90C2A	Thailand	MRA-202	Yes	
TM90C6A	Thailand	MRA-204	Yes	
TM91C235	Thailand	MRA-206	Yes	
T116	Thailand	S Thaitong/D Kyle	No	
TM327	Thailand	S Thaitong/D Kyle	Yes	
TD194	Thailand	S Thaitong/D Kyle	No	
PR145	Thailand	S Thaitong/D Kyle	Yes	R
TM335	Thailand	S Thaitong/D Kyle	No	
TM336	Thailand	S Thaitong/D Kyle	No	
TM343	Thailand	S Thaitong/D Kyle	No	
TM345	Thailand	S Thaitong/D Kyle	No	
TM346	Thailand	S Thaitong/D Kyle	No	
TM347	Thailand	S Thaitong/D Kyle	No	
TD203	Thailand	S Thaitong/D Kyle	Yes	R
TD257	Thailand	S Thaitong/D Kyle	Yes	R
TM-4C8-2	Thailand	S Thaitong/D Kyle	Yes	S
GA3	Thailand	S Thaitong/D Kyle	Yes	R
GH2	Thailand	S Thaitong/D Kyle	Yes	
MT/s1	Thailand	MRA-822	Yes	S
T2/C6	Thailand	MRA-818	Yes	
V1/S	Vietnam	MRA-176	Yes	R

*Mixed infections identified using PCR-based assays of 12 high-frequency SNPs.

### Variation in SNP diversity

**Figure 1:**
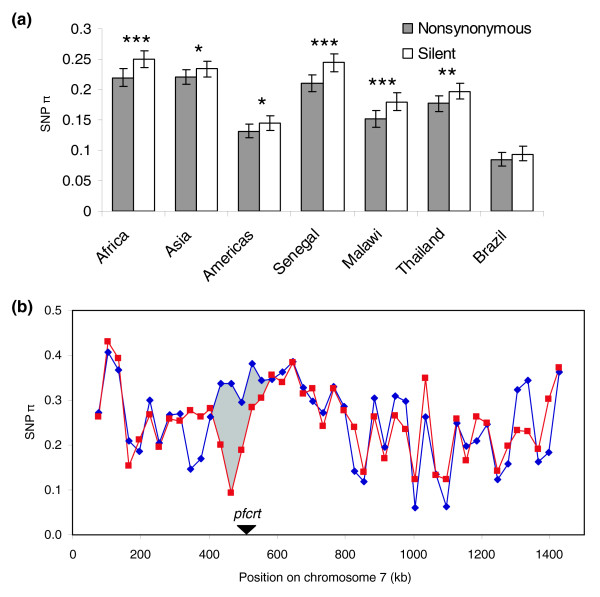
**Diversity at assayed SNPs (SNP π)**. **(a) **Nonsynonymous and silent SNP diversity by population. Significantly lower nonsynonymous SNP diversity (determined by bootstrapping) is indicated by asterisks: *P < 0.05; **P < 0.001; ***P < 0.0001. Error bars indicate 95% confidence intervals derived from bootstrapping. **(b) **SNP π on chromosome 7 for chloroquine resistant (red) and chloroquine sensitive (blue) samples. The disparity in diversity near 460 kb indicated with gray shading likely corresponds to a selective sweep associated with the *pfcrt *locus.

Overall SNP diversity at assayed loci varied by geographic region. Diversity was quantified using a statistic we term 'SNP π', defined as the average proportion of pairwise differences at assayed SNP loci within a defined population. At the continental scale, we measured the following SNP π values across all SNPs, with 95% confidence intervals indicated in parentheses: Africa = 0.234 (0.224-0.244); Asia = 0.227 (0.219-0.236); Americas = 0.14 (0.130-0.147). This ranking agrees with observations made using microsatellites [10], but differs from estimates obtained using 183 SNPs from chromosome 3 (Americas > Africa > Papua New Guinea > South-East Asia) [12]. At an intra-continental scale, we observed SNP π values of 0.227 (0.217-0.236) for Senegal, 0.165 (0.154-0.175) for Malawi, 0.187 (0.178-0.196) for Thailand, and 0.090 (0.081-0.098) for Brazil. For all defined populations, SNP diversity is higher for silent (synonymous or noncoding) SNPs than for nonsynonymous SNPs (Figure 1a), consistent with a greater proportion of nonsynonymous SNPs being subject to positive or negative natural selection.

We assessed the effect of selective sweeps on our SNP diversity data by examining the known selective sweep for chloroquine resistance around *pfcrt *on chromosome 7. We assayed the genotyped strains for resistance to chloroquine, and divided the Asian and African samples into chloroquine-resistant (CQR) and chloroquine-sensitive (CQS) groups. Separately calculating the SNP π values for each group, we found a clear signal of selection (permutation (see Materials and methods); *P *< 0.01) in the region of reduced diversity (located at approximately 460 kb) around *pfcrt *(Figure 1b), as might be expected following a selective sweep.

### Phylogenetic analysis

**Figure 2:**
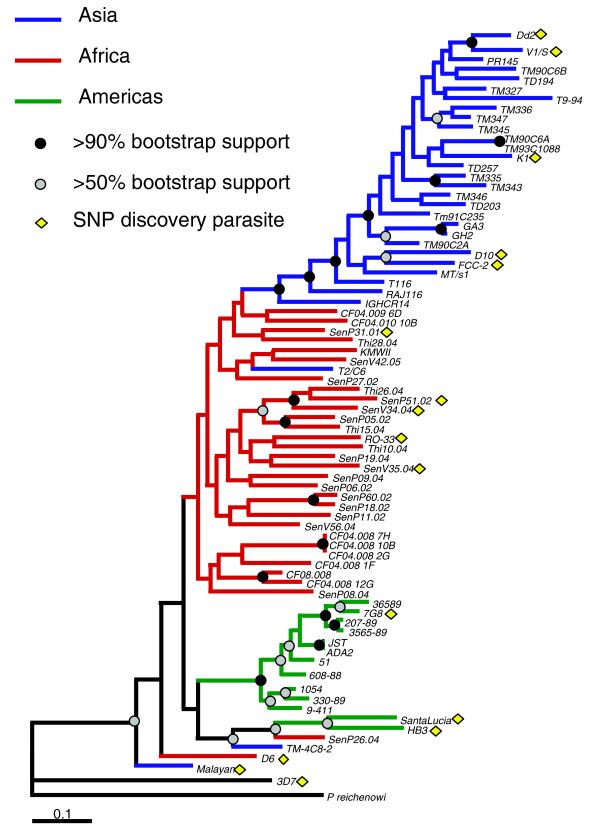
**Maximum likelihood phylogeny of global samples**. Blue, red, and green branches represent parasites from Asia, Africa, and the Americas, respectively. Parasites that were sequenced and thus were used for the discovery of SNPs are indicated by yellow diamonds. Nodes exhibiting bootstrap support levels of at least 50% or 90% are indicated by gray dots and black dots, respectively.

We examined the relatedness of the parasite isolates, and present a maximum likelihood phylogenetic tree rooted with *P. reichenowi *in Figure 2. This tree reflects strong bootstrap support for a clade composed largely of Asian samples, as well as a Brazilian clade, thereby offering strong evidence that *P. falciparum *is not a sexually panmictic species. The Senegal isolates (sample prefix = Sen or Thi) and Malawi isolates (sample prefix = CF) cluster together, although the clade they comprise does not exhibit strong bootstrap support. The HB3 and Santa Lucia samples (collected from Central America) are allied with the Brazilian samples, but curiously they also cluster strongly with the Senegal sample SenP26.04. SenP26.04, as well as other samples that do not cluster according to their sampling location (TM-4C8-2, D6, Malayan Camp, T2/C6) may represent strains with poor phylogenetic signal, cases of recent migration, or instances of cross-contamination in culture. The longer terminal branches of the Senegal isolates relative to the Brazilian or Asian isolates reflect the greater genomic diversity in that population, which is consistent with the higher rates of outcrossing and prevalence of infection observed in African populations.

SNP ascertainment bias can influence phylogenetic analyses if a limited number of reference strains are used to identify SNPs [16]. We performed SNP discovery from 16 strains of diverse geographic origins to reduce the potential for phylogenetic distortion. As Figure 2 illustrates, the SNP discovery strains (indicated by yellow diamonds) are distributed evenly across the major geographic clades, suggesting that this analysis is most likely not biased by geographically restricted SNP discovery. Our SNP set is also likely enriched for SNPs exhibiting high minor allele frequency (MAF), which are likely more phylogenetically informative than low MAF SNPs. Maximum likelihood phylogenetic analyses performed using the high MAF versus low MAF subsets of the SNP data yielded fundamentally congruent topologies (Additional data file 6), though the low-MAF tree exhibits compacted branch lengths and reduced bootstrap support.

The genomic reference strain 3D7 exhibits the longest branch of any ingroup taxon in the phylogenetic tree, resulting from the presence of 83 singleton SNPs in this lineage (142 if *P. reichenowi *is excluded). This abundance of singletons derives partially from bias in SNP ascertainment; 3D7 has been more deeply sequenced (12-18× coverage [17]) than any other strain, resulting in more variants unique to this strain being included on the array. (In sequence data, 380 SNPs on the array were singletons with the minor allele in 3D7, versus 146 singletons for HB3 and 53 singletons for Dd2, both of which were sequenced to 8× coverage [3].) The bias in SNP ascertainment may also contribute to the basal phylogenetic position of 3D7, near the *P. reichenowi *outgroup; 3D7 singletons with a derived (non-*P. reichenowi*) allele contribute to the long terminal branch length of 3D7, while 3D7 singletons with the ancestral (*P. reichenowi*) allele artificially enhance the evolutionary affinity of 3D7 and *P. reichenowi*.

### Population structure

We further analyzed the SNP data with two other population genetic analysis software packages, the programs Structure (v2.2.2) and SmartPCA. Structure uses a Bayesian approach to calculate the posterior probability of the number of populations sampled by a multilocus genotype dataset [18]. For our dataset the posterior probability asymptotes at 3 populations (Additional data file 7a), suggesting that continental boundaries between Asian, African, and American populations give rise to most of the population structure we can detect. Posterior probabilities of population membership for each of the samples (Additional data file 7b) are generally concordant with the phylogeny, and indicate that most samples sort unambiguously into expected groups according to continent of origin. 3D7 is assigned a 99.6% posterior probability of having derived from the African population, suggesting the isolate may have originated from an African population not sampled by the present dataset and/or temporal turnover in allele frequencies since 3D7 was collected almost 30 years ago.

We used SmartPCA [19] to perform a principal components analysis of the dataset, leading to very similar conclusions: southeast Asian, African and Brazilian samples form well-defined clusters, with the same few anomalous strains as in the other analyses (Additional data file 8). The one Papua New Guinea sample is on the edge of the southeast Asian cluster, and the Indian samples lie between the South-East Asian and African clusters. Preliminary principal components analyses that included SNPs in the region of the *pfcrt *selective sweep on chromosome 7 resulted in a heavy loading of signal in that region (data not shown), so SNPs from that region were excluded from the present analysis. Principal components analysis suggests two additional conclusions. First, it provides evidence for structure within the Brazilian population, consistent with a previous report [20] (Additional data file 8a); a larger sample size will be needed to confirm this suggestion. Second, it shows only a weak signal for shared ancestry between the two Central American parasites and the Brazilian cluster (in the first principal component), and strong signals of independent evolution (in the second and third principal components). This result cannot determine whether *P. falciparum *was introduced independently into the two regions, but it does suggest that there has been little gene flow between them.

Analysis of population divergence using the F_ST _statistic corroborates genetic differentiation at the continental scale and further finds significant genetic differentiation between the two African populations (Senegal versus Malawi F_ST _= 0.181). All pairwise population comparisons are significant by bootstrapping at *P *< 10^-4^, but the magnitude of F_ST _varies considerably among the comparisons. We found the greatest differentiation between Asian and American samples (F_ST _= 0.431), followed by Africa versus Americas (0.306), and Africa versus Asia (F_ST _= 0.236). The much greater genetic differentiation between American and Asian *P. falciparum *populations than American and African populations (bootstrapping; *P *< 10^-4^) supports a hypothesis of recent colonization of the Americas by African *P. falciparum *strains after the arrival of Europeans.

**Figure 3:**
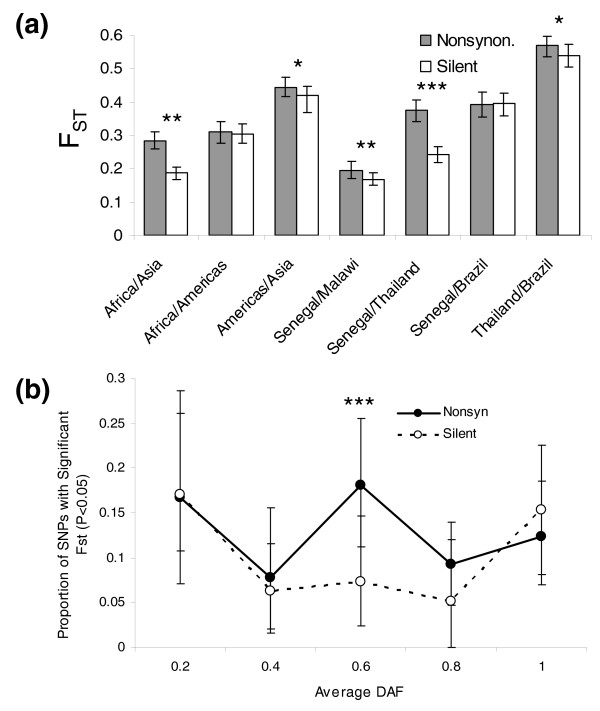
**Nonsynonymous and silent divergence (F_ST_)**. **(a) **Significantly greater nonsynonymous divergence (determined by bootstrapping) is indicated by asterisks: *P < 0.05; **P < 0.001; ***P < 0.0001. Error bars indicate 95% confidence intervals determined from bootstrapping. **(b) **Proportion of SNPs with significant Senegal versus Thailand F_ST _(*P *< 0.05) controlling for average derived allele frequency in Senegal and Thailand.

For all population comparisons except Senegal versus Brazil, we observe a greater F_ST _for nonsynonymous SNPs relative to silent SNPs (Figure 3a), suggesting that differential positive or negative selection may play a significant role in population differentiation. Alternatively, SNP ascertainment bias could potentially be differentially influencing the frequency spectra of nonsynonymous and silent SNPs and yielding an artifactual difference in F_ST_. Binning SNPs by average derived allele frequency (DAF) across Senegal and Thailand to control for this effect indicates that nonsynonymous SNPs still exhibit greater population structure than silent SNPs (Figure 3b), especially among SNPs with intermediate average DAF across those two populations. This observation holds true if SNPs from the densely sampled chromosome 7 are excluded from analysis (Additional data file 9), suggesting the phenomenon of enhanced nonsynonymous divergence is a genome-wide rather than localized effect.

**Figure 4:**
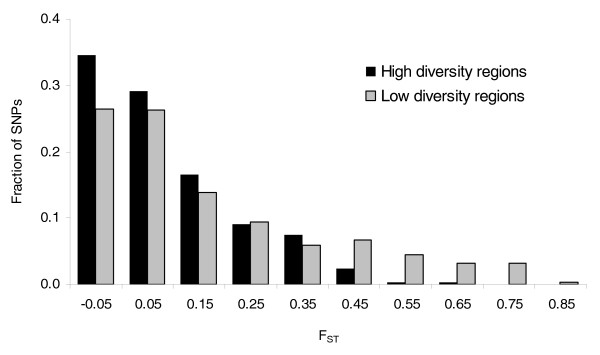
**Distribution of Thailand-Senegal divergence (F_ST_), plotted separately for markers in low diversity (SNP π < 0.005) and high diversity (SNP π > 0.005) windows**. Window size is 20 kb.

The importance of selection in shaping *P. falciparum *variation can be further seen by comparing divergence between populations with genetic diversity. Antigenic loci in *P. falciparum *are subject to diversifying selection, and often have extremely high diversity. We can expect the intense pressure for diversity to impede positive selection for other traits, and that high divergence between populations will, therefore, be uncommon near these loci. Indeed, we find that divergence (F_ST_) between the Senegal and Thailand populations is inversely correlated with *P. falciparum *diversity (as measured worldwide) at the same locus (Pearson's ρ = -0.17); high F_ST _SNPs are, in fact, almost absent from high diversity regions (Figure 4; Fishers exact test, *P *= 2 × 10^-6^). An additional factor, also related to selection, is probably operating here as well: loci that have experienced population-specific selective sweeps will have higher divergence with other populations as well as reduced diversity in that population, which will be reflected in the global diversity. Differential intensity of purifying selection between populations would be unlikely to yield a similar negative regional association between diversity and divergence, as relaxed purifying selection in a population would be expected to simultaneously and uniformly increase both divergence and diversity. Whatever the particular mechanism, the correlation illustrates the important role selection has in shaping population divergence in *P. falciparum*.

### Derived allele frequency spectra

**Figure 5:**
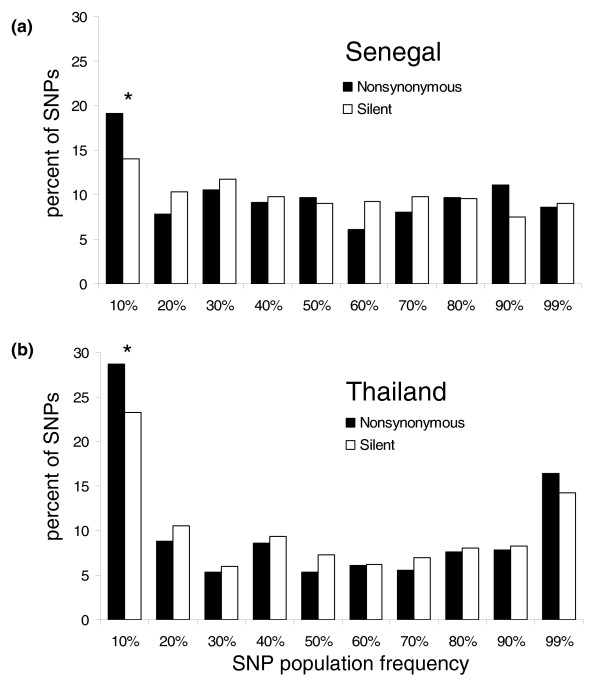
**Derived allele frequency spectra in (a) Senegal and (b) Thailand**. Bins exhibiting significant differences in frequency by Fisher's exact test between nonsynonymous and silent SNPs are indicated by asterisks: *P < 0.05).

Figure 5 illustrates the DAF spectra in Senegal and Thailand for all nonsynonymous and silent (noncoding and synonymous) SNPs for which the ancestral genotype could be inferred. SNPs were chosen for the array in part due to preliminary indications from comparative sequencing that they were not singletons, so these frequency spectra are likely enriched for higher-frequency variants and cannot be interpreted directly in comparison to neutral expectations. In both populations, there is a slight excess of low frequency (0-10%) nonsynonymous SNPs relative to silent SNPs (Fisher's exact test; Senegal *P *= 0.03, Thailand *P *= 0.05). This may be indicative of purifying selection maintaining slightly deleterious amino acid replacements at low population frequencies. The nonsynonymous and silent DAF spectra for Thailand show an abundance of high-frequency (90-99%) derived SNPs relative to Senegal. An excess of high-frequency derived alleles is an expected byproduct of genetic hitchhiking [21], whereby selective sweeps are 'interrupted' by recombination and leave partially linked alleles segregating at high frequencies instead of fixing them in the population [22].

Alternatively, the excess of SNPs exhibiting high DAF in Thailand could result from hidden population structure in the sample, where a derived allele is precluded from being classified as 'fixed' (100% frequency) by the inclusion in the sample of a few isolates belonging to a distinct population where the derived allele has not fixed. If this were the cause of the excess of high frequency derived alleles, we would expect the same few samples to exhibit an ancestral allele at multiple loci with high DAF. We find evidence to support this hypothesis. Though the Thailand samples T116, PR145, and TD257 are not phylogenetically distinct from the other Thailand samples (Figure 2) nor distinct by the F_ST _metric (F_ST _= 0.175, bootstrapping *P *= 0.69), these 3 strains (out of 23 in the population sample) exhibit an ancestral allele for approximately two-thirds of high DAF SNPs in both the nonsynonymous and silent data sets. Such a non-uniform distribution of ancestral alleles is unexpected from a freely mixing population. We conclude that hidden population structure, perhaps exacerbated by reduced outcrossing in Thailand *P. falciparum *populations, is responsible for the contrast in DAF spectra between Senegal and Thailand.

Though hidden population structure and SNP ascertainment bias may confound direct interpretation of DAF spectra in Senegal and Thailand, the DAF correlation between these two populations is more directly indicative of natural selection. Nonsynonymous SNPs exhibit a significantly weaker DAF correlation between Senegal and Thailand than silent SNPs (r^2 ^= 0.25 and 0.45, respectively; bootstrapping *P *< 10^-4^; Additional data file 10). Both nonsynonymous and silent derived allele frequencies are susceptible to bias from hidden population structure or other departures from demographic equilibrium, so the contrast between these two classes of SNPs is most likely due to population-specific natural selection.

**Figure 6:**
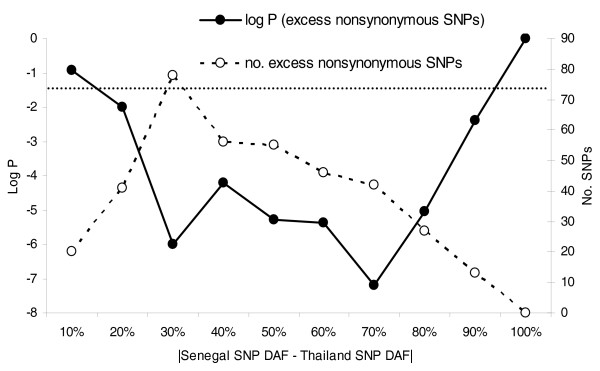
**Significance and count of excess nonsynonymous SNPs segregating at various disparities in frequency between Senegal and Thailand**. Statistical significance was evaluated in relation to synonymous SNP counts using Fisher's exact test. Horizontal dotted line indicates a *P*-value threshold of 0.05.

We can roughly estimate the proportion of nonsynonymous SNPs subject to differential positive or negative selection as a function of the disparity in derived allele frequency between Senegal and Thailand. If nonsynonymous and silent SNPs are subject to equal evolutionary pressures, demographic events, and ascertainment biases, then similar proportions of these two classes of SNPs should exhibit differences in allele frequency between the two populations that are above or below a threshold value. Deviations from this expectation are measurable by a Fisher's exact test applied to a 2 × 2 contingency table containing counts of the number of SNPs in each class above or below the threshold frequency disparity. The solid line in Figure 6 illustrates the statistical significance of the excess of observed nonsynonymous SNPs segregating with inter-population frequency disparities at least as divergent as the threshold values listed on the horizontal axis. The dashed line indicates the surplus of nonsynonymous SNPs above each frequency difference threshold, and may give a rough indication of the proportion of SNPs subject to selection in each class. The most statistically significant deviation from expectations occurs for SNPs with a disparity in population frequencies of at least 70% (bootstrapping *P *= 6.34 × 10^-4^). We find 55 nonsynonymous SNPs with frequency deviations above this threshold compared to only 13 synonymous mutations, from an original set of 598 nonsynonymous SNPs and 598 (by coincidence) silent SNPs that were polymorphic in one or both of these populations. By simple inference, then, 55 - 13 = 42 (7%) of the nonsynonymous SNPs in this class may be subject to selection, as represented by the dashed line in Figure 6. The largest observed excess of nonsynonymous SNPs (n = 78; 13%) was observed for a threshold of only 30% frequency disparity between the populations (bootstrapping *P *= 1.02 × 10^-6^), suggesting that even minor differences in nonsynonymous SNP allele frequencies among parasite populations may reflect the influence of natural selection.

### Linkage disequilibrium

**Figure 7:**
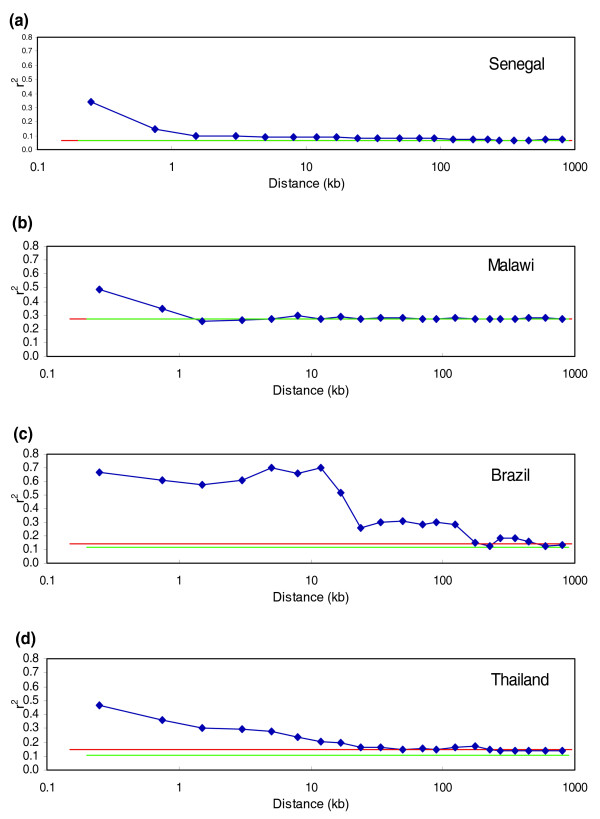
**Linkage disequilibrium, measured by r^2^, for each of the four population samples: (a) Senegal; (b) Malawi; (c) Brazil; (d) Thailand**. Plotted are the measured r^2 ^for linked markers (blue lines) and for unlinked markers (red lines), as well as the level of background LD expected because of small sample size (green lines).

Analysis of LD using the full set of genotyped SNPs reveals striking variation in the extent of LD across different populations. A greater extent of LD in Asian than in African populations has previously been reported [3,10,12], and our results (Figure 7) confirm that the difference is indeed large. Both the Senegal and the Malawi samples show essentially zero LD (above an artifactual background caused by small sample size) for markers separated by more than 1,000 base pairs. Modest but statistically significant LD extends to approximately 20 kb in the parasites from Thailand and to approximately 100 kb in the Brazilian sample. One contributor to the visible LD could be the known selective sweep on chromosome 7 (around the *pfcrt *gene), where our SNPs are concentrated. Removing that region (defined as extending from 300-600 kb along the chromosome, and containing 219 SNPs) did reduce r^2^, but only at very short distances and only by approximately 5%, suggesting that the selective sweep does not significantly bias allele frequencies otherwise analyzed. Assuming there has been no recent large-scale admixture in Thailand and Brazil, the longer LD can be attributed to their smaller effective population size and the reduced outcrossing in low-transmission locales.

The two non-African samples also showed a significant (bootstrapping; *P *< 10^-4^) amount of LD between unlinked markers, as determined by calculating r^2 ^between markers lying on different chromosomes. Correlation between alleles at unlinked markers is evidence for population structure within the data sample. Since accidental inclusion of closely related samples can produce the same effect, we removed one sample from the most closely related pair of Thai samples and redid the calculation; the change to measured LD was negligible. Population structure has previously been reported within South America [10] and within Brazil itself [20], but not previously for Thailand.

## Discussion

The results we present here on malaria population biology are important in that they confirm with significantly more data previous observations about the distribution of diversity in the *P. falciparum *parasite population, highlight the influence of natural selection on *P. falciparum *genomic diversity and divergence, and provide strong evidence that larger datasets will prove very useful in learning more about the biology of the parasite and the manifestation of disease. For example, though previous multilocus genotyping efforts in this parasite succeeded in documenting heterogeneous diversity levels and genetic differentiation among populations, the scale of the present analysis has enabled us to statistically distinguish the pattern of differentiation at nonsynonymous SNPs potentially subject to natural selection and synonymous or silent SNPs that are presumably closer to selective neutrality, but otherwise subject to similar effects from demographic effects and nearby selective sweeps. We observed higher F_ST _values for nonsynonymous SNPs than for silent SNPs in all population comparisons except Africa versus Americas (which exhibited highly similar values for both classes), which suggests a role for natural selection in the ongoing genetic differentiation of malaria populations in disparate geographic locales. Though a previous investigation observed a similar contrast in divergence between ten nonsynonymous SNPs at loci known to be associated with anti-malarial drug resistance and ten silent SNPs at housekeeping or unknown loci [23], we have demonstrated that nonsynonymous variation exhibits a distinct evolutionary profile across the genome, and not only at loci known to be subject to drug-mediated selection.

Both negative (purifying), and positive (directional/balancing) selection can increase population divergence if the efficacy of the selection differs geographically [24]. A recent study of human SNP data found that nonsynonymous SNPs were more likely than synonymous SNPs to exhibit extremes of both low divergence (attributed by the authors to purifying selection) and high divergence (attributed to positive selection) [25]. Our data show only half of this pattern, an overall trend for nonsynonymous SNPs to have higher divergence than silent ones. This pattern suggests that either localized positive selection or strong differences in selective constraints play a substantial role in shaping population differentiation in *P. falciparum* (the failure to detect the clearest signal of purifying selection, an excess of low-F_ST _nonsynonymous SNPs, may simply result from small sample size).

Even if negative selection plays an important role in nonsynonymous SNP divergence among *P. falciparum *populations, there are numerous potential population-specific environmental factors that could induce differentiation by positive selection. For example, population differentiation at nonsynonymous SNPs could result in variation among human host populations in HLA allelic composition, different *Anopheles *mosquito vectors, and/or heterogeneous application of anti-malarial therapeutics. Alternatively, some of the variant nonsynonymous SNPs may represent positively selected mutations that have arisen due to selection pressures that are present in both populations, but which have been prevented from spreading between the populations due to geographic barriers to gene flow. Deeper probing of these questions with expanded sample sets and denser SNP assays should illuminate whether these observed patterns of divergence and polymorphism are due to negative, positive, or balancing selection pressures.

Nonsynonymous SNPs that vary widely in frequency among *P. falciparum *populations warrant deeper follow-up investigations, as many may represent functional variations with important implications for public health or understanding of basic malaria biology. Nonsynonymous SNPs that differ even marginally in frequency among populations may be subject to selection (Figure 6). It should be noted that while the SNPs selected for inclusion in our pilot array are biased for a number of reasons, they are not expected to be enriched for variants subject to selection. Indeed, nonsynonymous SNPs in highly polymorphic subtelomeric gene families are known to be subject to strong immune selection, but are virtually absent among the SNPs on our array due to difficulties in recognizing and genotyping SNPs that are in close physical proximity. In all, only 12 of our SNPs were in clearly identified antigenic genes (as defined by Gene Ontology category).

The SNPs we profiled, consequently, may be expected to yield a conservative estimate of the degree to which segregating genetic variation in *P. falciparum *is subject to population-specific positive or negative natural selection. This broad survey of genetic diversity in *P. falciparum *stands in contrast to most previous analyses of variability in the parasite, many of which focused on antigenic loci such as *AMA-1 *[26,27] or *MSP-1 *[28] that are known to be subject to strong positive selection. While these population genetic analyses of antigens yield valuable information about the loci in question, the influence of strong host-mediated selective pressure erases typical patterns of genetic diversity and LD in the region of these genes, making difficult any accurate inferences about demographic history or population structure from the data. The very short range of detectable LD on chromosome 7 makes it likely that the profile of diversity we have observed is not dominated by one or more linked loci subject to strong selection (such as *pfcrt*), but instead that it reflects many independent, relatively unbiased local profiles of the interplay of natural selection and genetic drift.

## Conclusion

Our observations of large disparities in SNP diversity levels and LD magnitude, as well as significant selection-driven genetic differentiation among geographic populations of *P. falciparum*, underscore the extreme variability of the population biology and epidemiology of malaria [3,10,12]. Our data confirm that the greatest levels of genetic diversity and outcrossing occur in African populations of *P. falciparum*, likely as a result of greater incidence of infection (population size) and simultaneous infection of single hosts by multiple parasite lines (which facilitates sexual outcrossing in the mosquito). Consideration of such population factors will be important in interpreting observations of SNP diversity and selecting appropriate locations for genome-wide association studies, which require LD between SNP markers and causal loci for success. For example, if resistance to an anti-malarial drug arises in an Asian or American *P. falciparum *population, strong selection and low rates of outcrossing will likely leave a strong signal in the SNP diversity within those populations detectable through haplotype-based tests [29].

In summary, we have demonstrated the effectiveness and efficiency of the Affymetrix SNP genotyping array as a platform for high-throughput genotyping in the AT-rich *P. falciparum *genome, and have used a pilot version of this platform to perform the most comprehensive genome-wide genotyping analysis to date in this parasite. We are developing an expanded version of this array that will use probes to survey approximately 75,000 *P. falciparum *SNPs, as well as tiled probes to detect known and novel polymorphisms in dozens of other malaria genes of interest. We expect this high-density, genome-wide multilocus genotyping approach to have broad utility for investigations of basic malaria biology as well as for public health applications such as diagnostics and surveillance for drug resistance. Population genetic analyses that exploit regional variation in diversity or LD across the genome have already proven useful for re-identifying known drug-resistance loci [3,30-32], and will become more powerful tools for identifying genetic loci underlying traits of interest as further SNPs are discovered and incorporated into high-throughput genotyping platforms. Genome-wide association studies driven by such a tool will provide investigators with another avenue to identify the genes behind important disease phenotypes, and may help bridge some of the outstanding gaps in our knowledge of gene function and basic biology of the parasite.

## Materials and methods

### Genotyping array and hybridization

A standard Affymetrix array design was utilized, with each SNP interrogated by a set of fifty-six 25-mer nucleotide probes arranged in fourteen probe quartets (one 'perfect-match' and one 'mismatch' probe for each of the two alleles). SNPs identified through comparative sequencing [2-4] were selected for the inclusion on the Affymetrix array according to several considerations. Virtually all SNPs discovered on chromosome 7 (n = 2,153) were included in order to assess decay in LD as a function of distance from a known selective sweep on chromosome 7 at the *pfcrt *locus [11]. The remaining 843 SNPs were chosen by attempting to achieve uniform assay density over the remainder of the genome with SNPs that were non-singletons and achieved a high 'designability' rating using Affymetrix software. A list of validated SNP assays is included in Additional data file 5.

DNA was isolated from culture-adapted parasites (see 'Parasite samples' below) using standard 100/G protocols (Catalog number 13343, Qiagen, Germantown, MD, USA) and resuspended in TE (Tris EDTA) buffer for processing and hybridization. Genome-wide genotype data from the Affymetrix array were generated with the human 500 K array protocol but with a smaller hybridization volume of 125 μl owing to the smaller surface area of the malaria array.

Haploid SNP calls were made using several algorithms available in the Affymetrix Power Tools (apt-1.8.5): DM, BRLMM, and BRLMM-P. Default confidence thresholds were employed for all algorithms (*P *= 0.05 for BRLMM-P, 0.50 for BRLMM, 0.30 for DM). Genotype calls for 16 reference arrays were then compared against known sequencing data for the same strains [2-4]. SNPs that showed any discordance with sequencing data and SNPs that did not have overlapping calls for both alleles were discarded and only the remaining SNPs were used for analysis. The BRLMM-P data set was used for all analyses in this paper, as it exhibited the most perfectly concordant SNPs (1,638) and a much higher call rate than the other algorithms (Additional data file 1).

### Parasite samples

Parasites were obtained from the Malaria Research and Reagent Resource Repository (MR4, ATCC, Manassas, Virginia) or additional sources noted (Table 1). MR4 isolates were provided by the following depositors: WE Collins (MRA-362); DE Kyle (MRA-159, MRA-176, MRA-202, MRA-204, MRA-205, MRA-206, MRA-207, MRA-285); LH Miller and D Baruch (MRA-330); X Su (MRA-818, MRA-821, MRA-822); W Trager (MRA-733); D Walliker (MRA-151, MRA-152, MRA-153, MRA-200); TE Wellems (MRA-155, MRA-156); and Y Wu (MRA-201). Patient samples were obtained as part of ongoing studies in Senegal and Malawi described elsewhere in accordance with human subject guidelines. Parasites were cultured by standard methods and nucleic acids were obtained using Qiagen genomic-tips (100/G, Qiagen). *P. reichenowi *DNA was the generous gift of John Barnwell.

### Population genetic analyses

All population genetic analyses were conducted using SNPs that yielded 100% accurate genotyping results for both the major and minor assayed alleles, and which yielded calls in at least 80% of the population samples hybridized to the array. Measurements of population diversity were carried out using the 'SNP π' statistic, which was calculated as the average number of pairwise differences at assayed SNPs between all members of a sample. Population divergence was measured using the F_ST _statistic, calculated with the method of Hudson, Slatkin and Maddison [33]. Statistical significance of F_ST _and SNP π comparisons, as well as 95% confidence intervals for both statistics, were calculated using nonparametric bootstrapping. We also employed Structure 2.2 software [18] to examine parasite population structure. We conducted analyses twice for K values 2-5. Each analysis was allowed to run 100,000 iterations, with 50,000 burn-in cycles. An admixture model was used for all Structure analyses. Global nucleotide diversity (π) was calculated using the sequencing data in [3]. For this analysis, high diversity was defined as π > 0.005.

The maximum likelihood phylogenetic analysis was conducted using PHYML software [34] on a BIONJ [35] starting tree. An HKY85+Γ model of substitution was employed and 1,000 bootstrap replicates were performed. All model parameters were estimated from the data.

Population-specific DAF spectra are based on only those SNPs that are not fixed in a given population to reduce spectrum distortion from SNPs that were born in another population following. Consequently, slightly different SNP sets are used in each population. DAFs were estimated for 360 nonsynonymous and 399 silent SNPs in Senegal, and 397 nonsynonymous and 387 silent SNPs in Thailand.

### Linkage disequilibrium

LD was studied within each of the four populations with enough samples: Thailand, Malawi, Senegal and Brazil. For this analysis, two nominally Thai samples (T2/C6 and TM-4C8-2) were dropped that clustered with other populations in the phylogenetic analysis, and only parasites representing single genomes were included in the analysis. Missing genotypes were not imputed. The widely used LD statistic r^2 ^was calculated within each population for all pairs of SNPs sharing the same chromosome; pairs were binned by distance and averaged within each bin. The level of LD between unlinked markers was estimated by calculating r^2 ^between all pairs of SNPs on different chromosomes. To determine the bias caused by small sample size, the unlinked calculation was repeated, with the change that for each pair of SNPs, the genotype for one was taken from one strain while the genotype for the second was taken from another strain. This background value of r^2 ^was calculated separately for the possible pairs of different strains and then averaged. The statistical significance of the observed LD between unlinked markers was estimated by calculating r^2 ^under bootstrap re-sampling of unlinked markers; this provides an estimate of the statistical uncertainty on unlinked r^2 ^and an overestimate of the uncertainty on background LD. In 10,000 permutations, the unlinked value was always much higher (>10 standard deviations) than the background value.

### Drug sensitivity

Chloroquine diphosphate-sensitivity assays were performed as part of an ongoing screen of established *P. falciparum *strains against antimalarial drugs in clinical use. Parasites selected for the assays were derived from the same isolates that furnished DNA for the genotyping studies. Parasites were grown and sorbitol-synchronized to the ring stage by standard methods, and 72-hour drug responsiveness was measured using a DAPI DNA-staining assay in a 384-well plate format as previously described [36]. Dose-response curves were generated using nonlinear-regression fit with variable slope (Prism 5; GraphPad Software, San Diego, CA, USA) and IC50s for chloroquine calculated. For the purposes of this paper, chloroquine resistance was defined as IC50 >25 nM, with the sensitive 3D7 and resistant Dd2 strains exhibiting IC50s of 9 and 55 nM, respectively. Evidence for selection for chloroquine resistance was evaluated using a sliding window (of three consecutive 30 kb bins) test on chromosome 7, calculating the statistic $\frac{\left(\pi_{CQS}-\pi_{CQR}\right)}{\left(\pi_{CQS}+\pi_{CQR}\right)}$ (where π is the SNP π value) for each window. Significance was estimated by permuting the bins and counting the fraction of cases that yielded a value of this statistic higher than seen in the data around *pfcrt*.

## Abbreviations

BRLMM: Bayesian Robust Linear Modeling using Mahalanobis Distance; DAF: derived allele frequency; DM: Dynamic Modeling; LD: linkage disequilibrium; MAF: minor allele frequency; SNP: single nucleotide polymorphism.

## Authors' contributions

DEN and SFS designed experiments, performed population genetic analyses and wrote the paper. SKV designed experiments, prepared samples, and wrote the paper. DP performed SNP calling and analysis of raw genotyping data. PM supervised SNP calling and raw data analysis. DAM and AL helped with parasite cultures and consulted on project outcomes. DR helped with parasite culture. RD extracted and prepared DNA for hybridization. NH and CG hybridized samples to the array. JFC and ET performed drug phenotyping assays. NS-T created DNA libraries. OS, DN, ON, SM, MF, SM, AD, and CC helped with sample collection. RCW coordinated project flow and supervised data collection. DLH consulted on population genetic analysis. BWB supervised and advised on data collection. ESL consulted on project outcomes. PCS designed experiments, consulted on population genetic analysis and wrote the paper. DFW designed experiments, coordinated all efforts, supervised the project at all levels, consulted on project outcomes and wrote the paper. All authors read and approved the final manuscript.

## Additional data files

The following additional data are available with the online version of this paper. Additional data file 1 is a histogram of SNP call rates. Additional data file 2 is discussion of array performance in the presence of human DNA and malaria DNA from mixed (non-clonal) infections. Additional data file 3 is a figure depicting array performance with mixed malaria genotypes. Additional data file 4 is a figure depicting array performance in the presence of human DNA. Concordance with known genotype can be improved using more stringent confidence cutoff values with the BRLMM-P calling algorithm. Additional data file 5 is a table illustrating the genomic location and genotype data for SNPs assayed on the array with a call rate of at least 80%. Additional data file 6 contains figures depicting maximum likelihood phylogenies constructed from high MAF or low MAF subsets of the data. Additional data file 7 contains figures depicting Structure analysis results. Additional data file 8 contains results from principal components analysis of population data using SmartPCA. Additional data file 9 contains a figure depicting the proportion of silent and nonsynonymous SNPs outside chromosome 7 with significant Senegal vs Thailand F_ST _(bootstrapping P < 0.05), controlling for average derived allele frequency in Senegal and Thailand. Additional data file 10 is a figure illustrating the nonsynonymous and silent SNP DAF correlation between Senegal and Thailand.

## Supplementary Material

Additional data file 1 Lines indicate the number of SNPs exhibiting various call rates using the DM, BRLMM, and BRLMM-P SNP calling algorithms. BRLMM-P SNP calls were used for analysis. Click here for file

Additional data file 2 Array performance in the presence of human DNA and malaria DNA from mixed (non-clonal) infections. Click here for file

Additional data file 3 Reported results are for SNP loci known to exhibit different alleles between the HB3 and Dd2 lines. The highest proportion of heterozygous calls was observed for the even (1:1) mixture of malaria. Click here for file

Additional data file 4 Concordance with known genotype can be improved using more stringent confidence cutoff values with the BRLMM-P calling algorithm. Click here for file

Additional data file 5 Genomic location and genotype data for SNPs assayed on the array with a call rate of at least 80%. Click here for file

Additional data file 6 **(a) **High MAF (MAF > 0.25) topology. **(b) **Low MAF (MAF < 0.25) topology. Nodes exhibiting bootstrap support levels of at least 50% or 90% are indicated by gray dots and black dots, respectively. Bootstrap support and branch length differ between the topologies, but the American and Asian parasites form congruent clades. Click here for file

Additional data file 7 **(a) **Plot of the likelihood of the genotyping data given that the samples derive from K = 1-5 populations. **(b) **Plot of the posterior probability of population membership for each sample hybridized to the array, assuming three underlying populations. Click here for file

Additional data file 8 **(a) **First two principal components for Brazil data; clusters suggest population structure. **(b) **First two components for worldwide data set. **(c) **First and third components for worldwide data set. Click here for file

Additional data file 9 Significantly greater nonsynonymous divergence (determined by bootstrapping) is indicated by asterisks: **P < 0.001. Click here for file

Additional data file 10 Nonsynonymous and silent SNP DAF correlation between Senegal and Thailand. Click here for file
