# Supplementary figures and images for: Genome-wide SNP genotyping highlights the role of natural selection in Plasmodium falciparum population divergence
Source: Genome Biol. 2008 Dec 15;9(12):R171. doi: 10.1186/gb-2008-9-12-r171 (PMC2646275; doi:10.1186/gb-2008-9-12-r171)

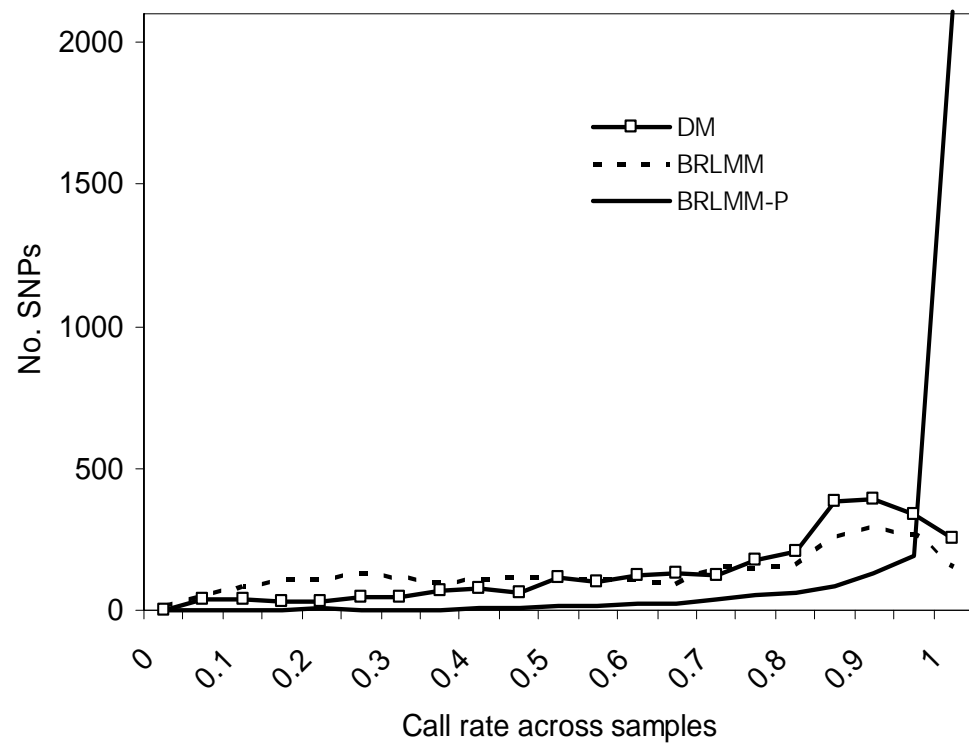

Supplement: Additional data file 1 — Lines indicate the number of SNPs exhibiting various call rates using the DM, BRLMM, and BRLMM-P SNP calling algorithms. BRLMM-P SNP calls were used for analysis. [file gb-2008-9-12-r171-S1.pdf]

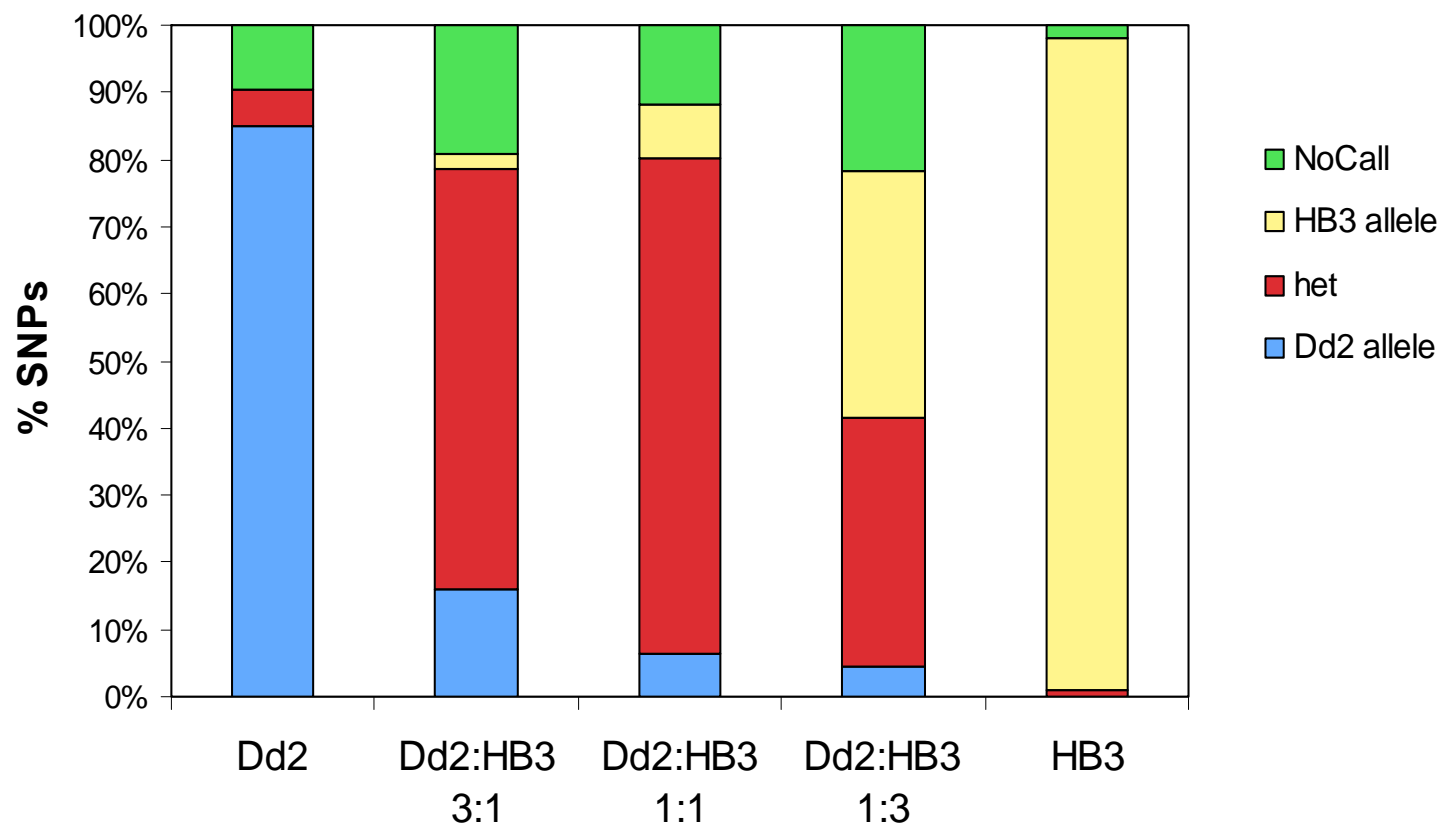

Supplement: Additional data file 3 — Reported results are for SNP loci known to exhibit different alleles between the HB3 and Dd2 lines. The highest proportion of heterozygous calls was observed for the even (1:1) mixture of malaria. [file gb-2008-9-12-r171-S3.pdf]

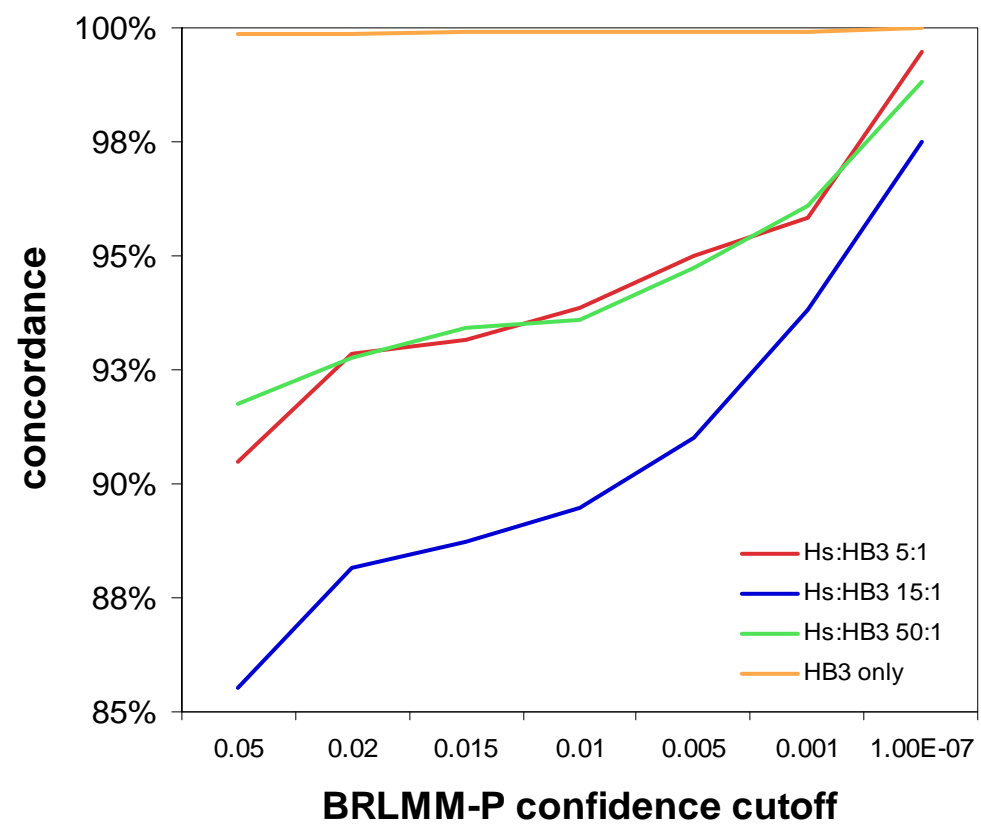

Supplement: Additional data file 4 — Concordance with known genotype can be improved using more stringent confidence cutoff values with the BRLMM-P calling algorithm. [file gb-2008-9-12-r171-S4.pdf]

A

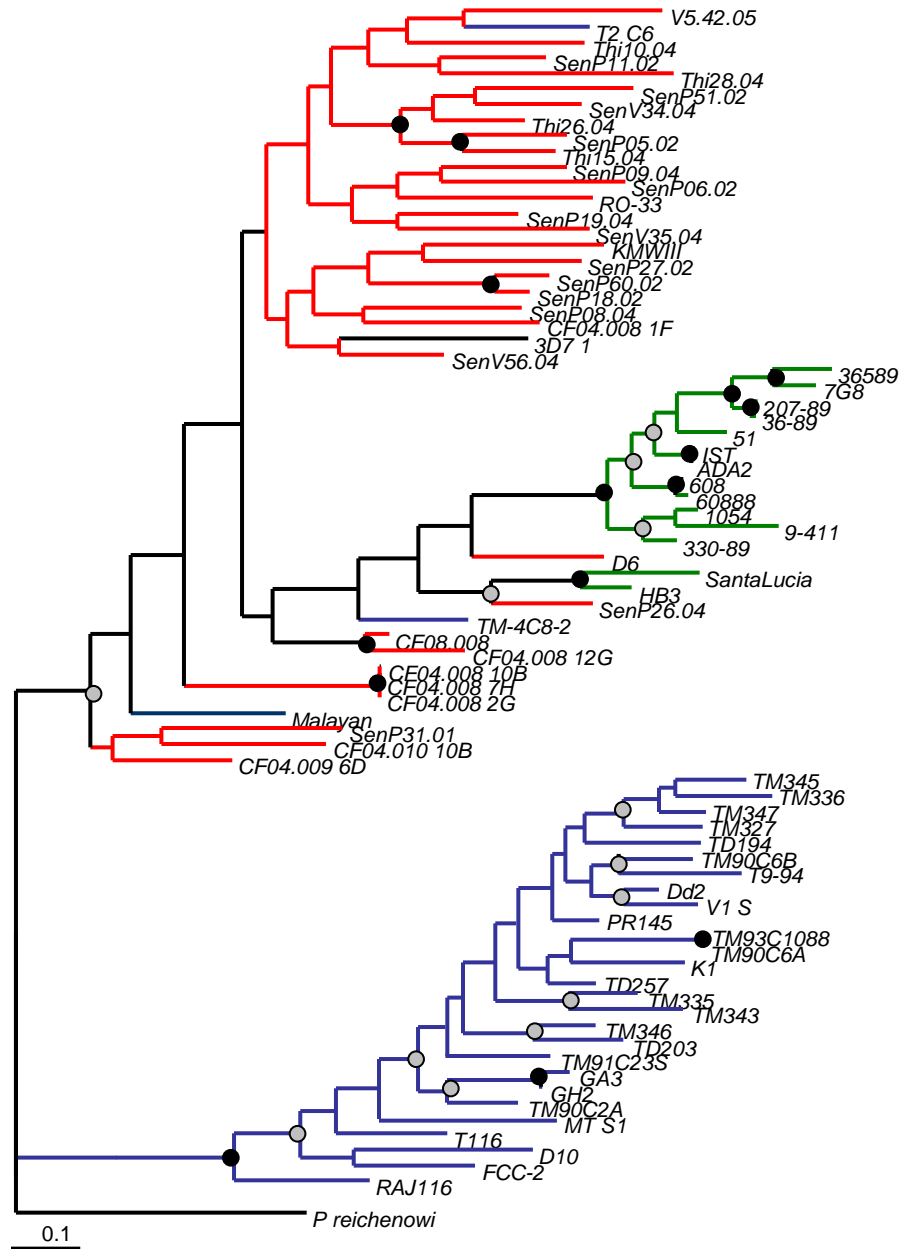

B

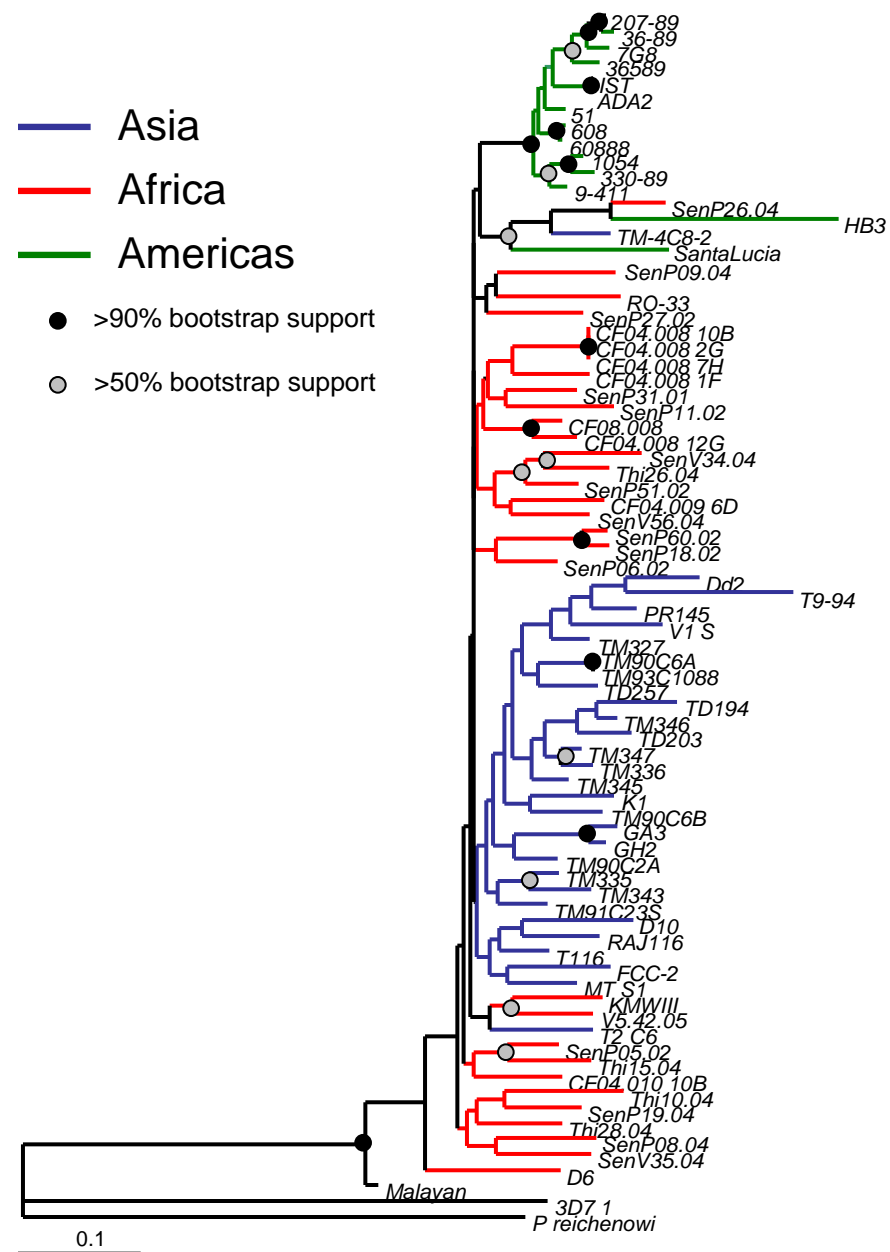

Supplement: Additional data file 6 — (a) High MAF (MAF > 0.25) topology. (b) Low MAF (MAF < 0.25) topology. Nodes exhibiting bootstrap support levels of at least 50% or 90% are indicated by gray dots and black dots, respectively. Bootstrap support and branch length differ between the topologies, but the American and Asian parasites form congruent clades. [file gb-2008-9-12-r171-S6.pdf]

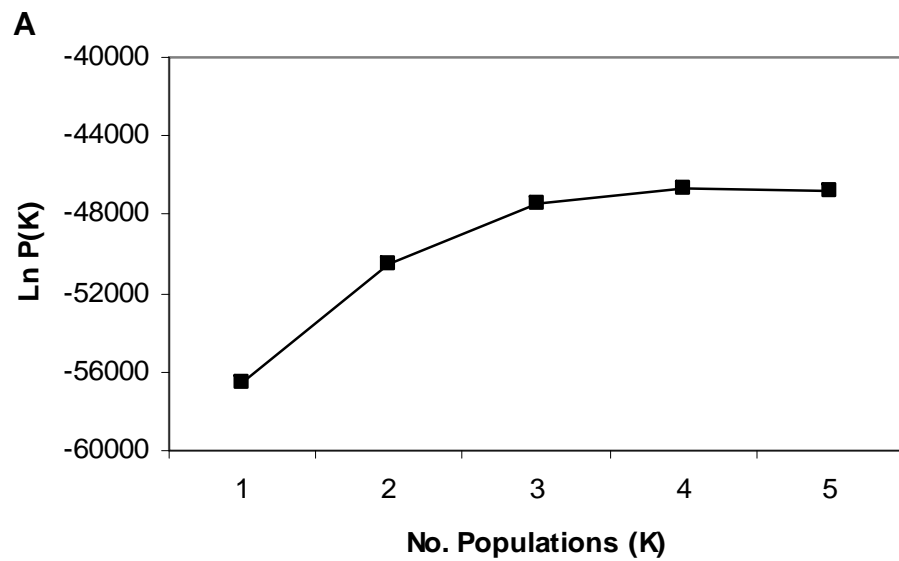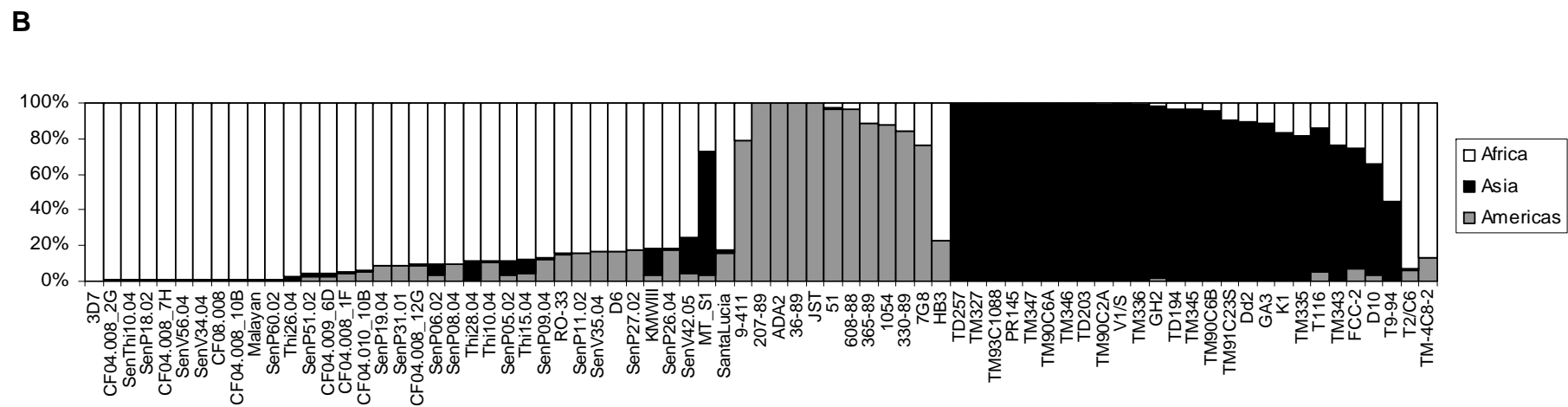

Supplement: Additional data file 7 — (a) Plot of the likelihood of the genotyping data given that the samples derive from K = 1-5 populations. (b) Plot of the posterior probability of population membership for each sample hybridized to the array, assuming three underlying populations. [file gb-2008-9-12-r171-S7.pdf]

**A**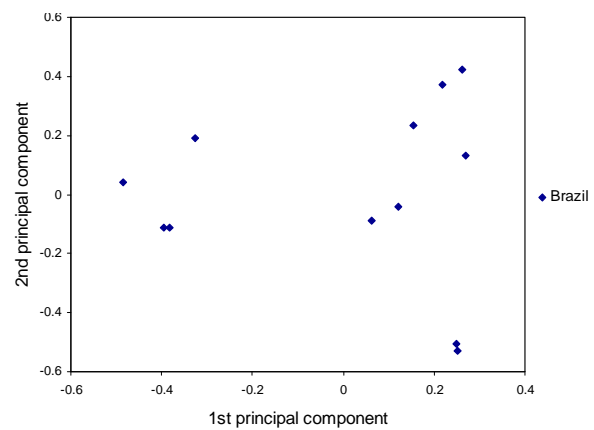**B**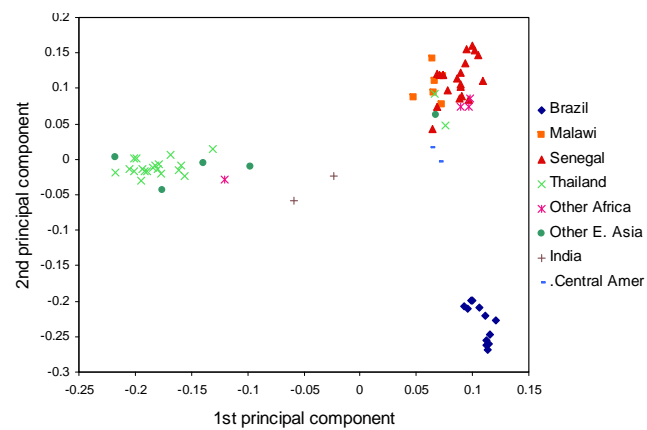**C**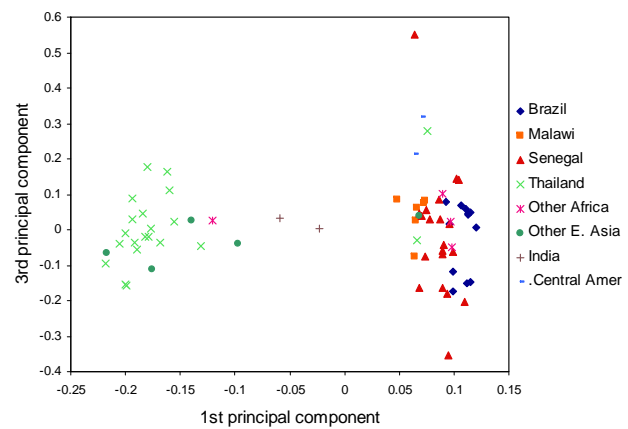

Supplement: Additional data file 8 — (a) First two principal components for Brazil data; clusters suggest population structure. (b) First two components for worldwide data set. (c) First and third components for worldwide data set. [file gb-2008-9-12-r171-S8.pdf]

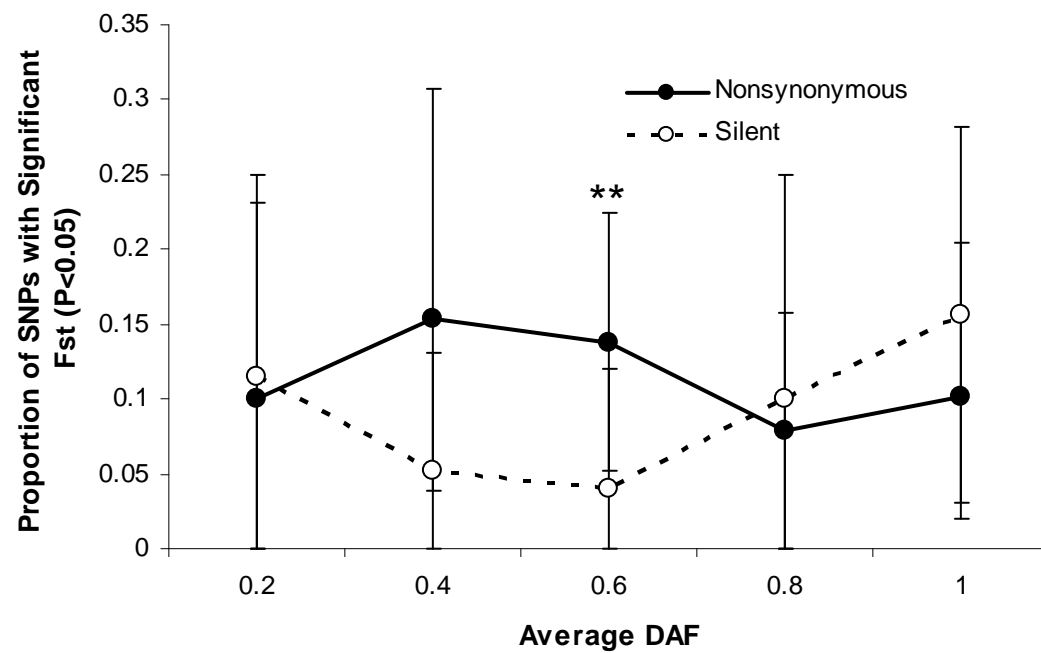

Supplement: Additional data file 9 — Significantly greater nonsynonymous divergence (determined by bootstrapping) is indicated by asterisks: **P < 0.001. [file gb-2008-9-12-r171-S9.pdf]

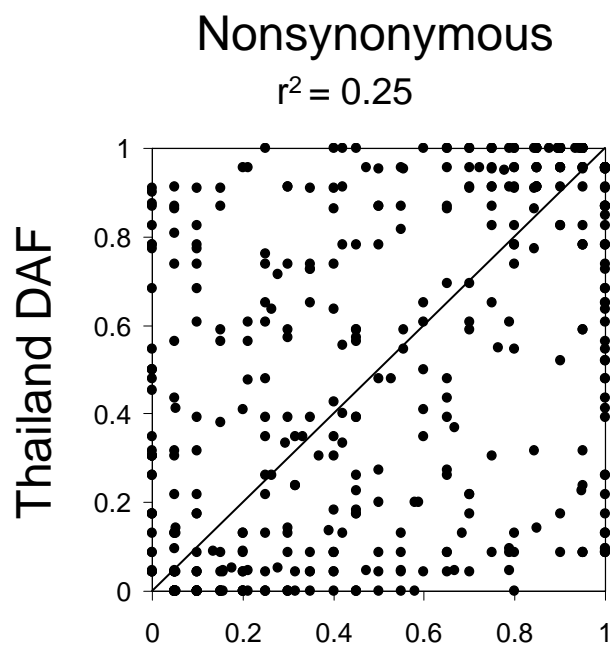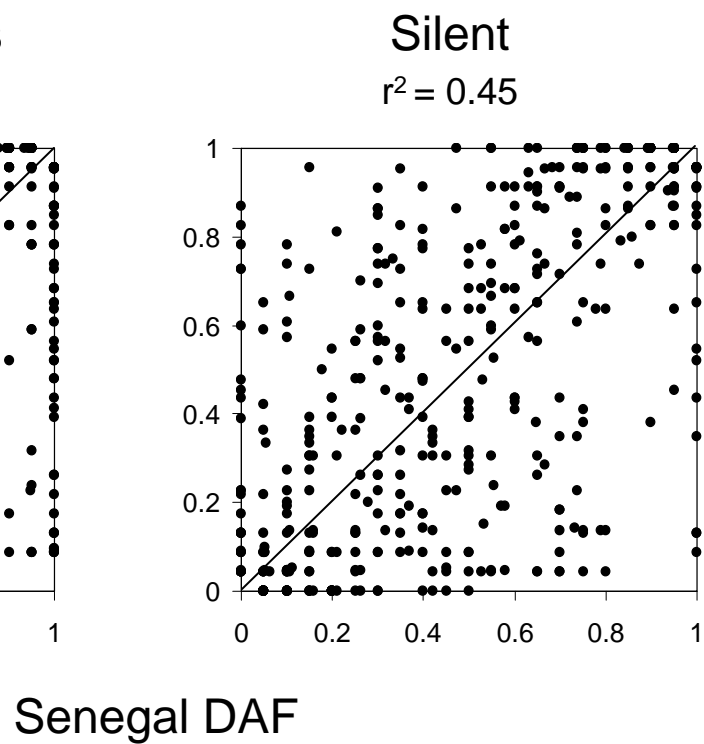

Supplement: Additional data file 10 — Nonsynonymous and silent SNP DAF correlation between Senegal and Thailand. [file gb-2008-9-12-r171-S10.pdf]
