# Supplementary material for: Genome-wide SNP genotyping highlights the role of natural selection in Plasmodium falciparum population divergence
Source: Genome Biol. 2008 Dec 15;9(12):R171. doi: 10.1186/gb-2008-9-12-r171 (PMC2646275; doi:10.1186/gb-2008-9-12-r171)
Supplement: Additional data file 2 — Array performance in the presence of human DNA and malaria DNA from mixed (non-clonal) infections. [file gb-2008-9-12-r171-S2.doc]

**Supplemental Results: Array Performance**

We hybridized mixtures of genomic DNA from different strains to the chip to assess its performance at recognizing heterozygous bases in mixed infections. We assessed chip performance for mixtures of genomic DNA from the HB3 and Dd2 lines in ratios of 3:1, 1:1, and 1:3, respectively, relative to pure genomic DNA preparations from these two lines. Additional data file 3 illustrates the impact of mixed genotypes on call rate and concordance for those SNPs known to differ between the two component strains. The chip was most successful at calling heterozygous bases when equal amounts (1:1) of DNA were present from the two strains. In that case, approximately 70% of heterozygous SNPs were correctly identified as such, and with the remainder of homozygous calls approximately equally split between the two component genotypes.

Chip performance with uneven ratios of Dd2 and HB3 genomic DNA (1:3 and 3:1) yielded a lower proportion (35-60%) of heterozygous base calls, and homozygous calls were heavily biased towards the dominant genotype in the mixture. These results indicate that this genotyping platform reacts to heterozygosity in a predictable manner, and that mixed infections containing component strains at very small fractions (<10%) may reflect the genotype of the dominant strain at the vast majority of genotyped loci.

We also hybridized *P. falciparum* genomic DNA to the chip in the presence of human DNA contamination. Additional data file 4 illustrates that a reduction in genotyping accuracy occurs with human:malaria DNA mixture ratios of 5:1, 15:1, and 50:1. Overall concordance with known malaria genotype across 1,638 SNPs drops from close to 100% with pure malaria DNA samples to 85-92% in the presence of human DNA. The proportion of human DNA in the sample used for hybridization does not appear to influence the overall concordance, as mixtures with 5:1 and 50:1 human:malaria DNA concentration ratios yielded similar results. Tuning the BRLMM-P statistical threshold for making genotype calls does indeed improve concordance to known malaria genotype for all three ratios of human:malaria DNA mixtures, though overall call rate suffers as a consequence. For example, to achieve a concordance rate of at least 95%, call rate drops to 57-62%. Though the loss in call rate is costly, this finding is encouraging in that it indicates that the deleterious consequences of human DNA in samples result from lower intensity binding of human DNA samples, and that the issues can be remedied by manipulation of the calling algorithm.
